# Cord Blood Mercury and Early Child Development: Effects of the World Trade Center

**DOI:** 10.1289/ehp.0800155

**Published:** 2009-01

**Authors:** José G. Dórea

**Affiliations:** Faculty of Health Sciences, Universidade de Brasilia, Brasilia, Brazil, E-mail: dorea@rudah.com.br

Lederman et al. (2008) assessed Psychomotor Development Index (PDI) and Verbal and Full Intelligence Quotient Scores (VFIQS) (at 36 months of age for PDI and 48 months for VFIQS) as a function of prenatal mercury exposure (corrected for maternal fish intake during pregnancy) resulting from potential exposure after the World Trade Center (WTC) disaster. This timely and interesting study took into consideration maternal variables known to influence cord Hg (possibly reflecting fish consumption) and also controlled for most of the known maternal characteristics that could affect neurodevelopmental outcomes. However, two of the most important variables in the context of infant exposure and neurodevelopment were left out of the model: early (pregnancy and postnatal) thimerosal-Hg exposure and the mode of feeding (Dórea 2007).

The children enrolled in the study were delivered at term in Lower Manhattan (New York, NY, USA) after 11 September 2001. Coincidently, around this time, the U.S. health authorities decided to withdraw thimerosal (as a preservative) from infants’ vaccines (Geier and Geier 2005). This decision generated controversy that impacted immunization and also demanded epidemiologic studies to evaluate the neurotoxic consequences (Thompson et al. 2007). Therefore, Lederman et al.’s (2008) study cohort probably contained not only children immunized with thimerosal-containing vaccines (TCV) but also some mothers using products containing thimerosal during pregnancy (e.g., Rh-negative mothers taking anti-RhoD immune globulins) (Geier et al. 2008).

Although TCV are no longer used in the United States and other developed nations, they are used elsewhere in underdeveloped countries, exposing infants to varied ethyl mercury (EtHg) concentrations (depending on the vaccine manufacturer) and a wide range of doses, depending on the infant’s weight (Dórea and Marques 2008). This controversial issue (safety of early exposure to EtHg and neurodevelopment) has only recently been considered in epidemiologic studies of U.S. children (Thompson et al. 2007). Although the beneficial effect of (maternal) fish consumption is a concept emerging in most recent studies that have explored the neurodevelopment of children, the few studies of TCV’s effects on neurodevelopment have been inconclusive in the United States. The most recent study neither adjusted for breast-feeding nor for additional prenatal methylmercury (MeHg) exposure from maternal fish consumption, but they attributed the conflicting outcomes to chance (Thompson et al. 2007). However, the additional EtHg exposure from postnatal TCV may contribute to a relative increase in infant hair-Hg (Marques et al. 2007). Coupled with that, the Faroese cohort studies (Grandjean et al. 1995; Jensen et al. 2005) cited by (Lederman et al. 2008) to demonstrate prenatal MeHg effects on neurobehavior have also noted the positive and confounding effects of breast-feeding.

We do not know the threshold of TCV–EtHg effects on neurodevelopment, but we do know that children born prematurely and those who were not breast-fed are at increased risk of having lower neurocognitive achievement (Kramer et al. 2008). Therefore, performance end points such as PDI and VFIQS could be affected by many intervening variables such as breast-feeding (and co-exposure to other toxic substances during brain growth and functional differentiation); these variables act mostly on the outcome, not necessarily on the level of Hg exposure. The rate of growth of the human brain is faster in the postnatal than in the antenatal period. At birth, the infant brain is 25.6% of the adult size, but it attains 50% of the adult size during the first 6 months (Passingham 1985); therefore, *ex utero* maternal nurturing (breast-feeding for 24 weeks) is more efficient than *in utero* (pregnancy, 36 weeks) in promoting brain growth concomitant with structural and functional differentiation. Unquestionably, prenatal and perinatal neuroactive substances can impact the central nervous system (CNS), affecting neuro-development at later times. Therefore, critical windows of CNS vulnerability that modulate the course of neurodevelopment (structural and functional) and that depend on maternal nurturing extend from late pregnancy to the postnatal period defined by breast-feeding. This implies that the interactions of cord Hg (fish intake and environmental Hg) and nonmeasured confounders (feeding mode and EtHg exposure) are difficult, but essential, in the evaluation of early neurocognitive achievements.

If Lederman et al. (2008) had included breast-feeding and EtHg exposure in their the statistical model, we could observe their proportional weight (if any) in the PDI and VFIQS outcomes. Regardless of model, this is still an interesting and important study. However, accounting for all sources of Hg exposure—and confounders such as breast-feeding—can bridge the gap between pragmatic vaccinology and conventional toxicology. TCV and early (and serial) exposure to EtHg may soon become an issue in other countries.
